# Efficacy and tolerability of regorafenib in pretreated patients with progressive CNS grade 3 or 4 gliomas

**DOI:** 10.1007/s11060-022-04066-9

**Published:** 2022-06-18

**Authors:** Jan-Michael Werner, Lena Wolf, Caroline Tscherpel, Elena K. Bauer, Michael Wollring, Garry Ceccon, Martina Deckert, Anna Brunn, Roberto Pappesch, Roland Goldbrunner, Gereon R. Fink, Norbert Galldiks

**Affiliations:** 1 Deptartment of Neurology, Faculty of Medicine and University Hospital Cologne, University of Cologne, Kerpener St. 62, 50937 Cologne, Germany; 2 Institute of Neuroscience and Medicine (INM-3), Research Center Juelich, Leo-Brandt-St. 5, 52425 Juelich, Germany; 3 Institute. of Neuropathology, Faculty of Medicine and University Hospital Cologne, University of Cologne, Cologne, Germany; 4 Center of Integrated Oncology (CIO), Universities of Aachen, Bonn, Cologne, and Düsseldorf, Cologne, Germany; 5 Institute of Pathology, Faculty of Medicine and University Hospital Cologne, University of Cologne, Cologne, Germany; 6 Deptartment of General Neurosurgery, Faculty of Medicine and University Hospital Cologne, University of Cologne, Cologne, Germany

**Keywords:** Multikinase inhibitor, Glioblastoma, Astrocytoma, Oligodendroglioma

## Abstract

**Background:**

The phase 2 REGOMA trial suggested an encouraging overall survival benefit in glioblastoma patients at first relapse treated with the multikinase inhibitor regorafenib. Here, we evaluated the efficacy and side effects of regorafenib in a real-life setting.

**Methods:**

From 2018 to 2021, 30 patients with progressive WHO CNS grade 3 or 4 gliomas treated with regorafenib (160 mg/day; first 3 weeks of each 4-week cycle) with individual dose adjustment depending on toxicity were retrospectively identified. Side effects were evaluated according to the Common Terminology Criteria for Adverse Events (version 5.0). MRI was obtained at baseline and after every second cycle. Tumor progression was assessed according to RANO criteria. After regorafenib initiation, the median PFS and OS were calculated.

**Results:**

The median number of treatment lines before regorafenib was 2 (range 1–4). Most patients (73%) had two or more pretreatment lines. At first relapse, 27% of patients received regorafenib. A total of 94 regorafenib cycles were administered (median 2 cycles; range 1–9 cycles). Grade 3 and 4 side effects were observed in 47% and 7% of patients, respectively, and were not significantly increased in patients with two or more pretreatments (P > 0.05). The most frequent grade 3 or 4 side effects were laboratory abnormalities (62%). PFS was 2.6 months (range 0.8–8.2 months), and the OS was 6.2 months (range 0.9–24 months).

**Conclusions:**

In patients with progressive WHO grade 3 or 4 gliomas, predominantly with two pretreatment lines or more, regorafenib seems to be effective despite considerable grade 3 or 4 side effects.

## Introduction

Treatment options in patients with WHO grade 3 or 4 glioma at progression include most frequently resection, re-irradiation, alkylating chemotherapy (e.g., concept of “re-challenge”), antiangiogenic therapy, other targeted therapies, or combinations thereof [1]. In the broad spectrum of targeted therapy, regorafenib is an orally available small molecule multikinase inhibitor targeting signaling pathways that drive angiogenesis, oncogenesis, and tumor microenvironment maintenance [2, 3]. The targets of regorafenib include the vascular endothelial growth factor 1–3, angiopoietin-1 receptor, proto-oncogene c-Kit, Ret proto-oncogene, Raf-1 proto-oncogene, platelet-derived growth factor receptor, fibroblast growth factor receptor, and v-Raf murine sarcoma viral oncogene homolog B (BRAF) [2, 3].

The randomized phase 2 REGOMA trial demonstrated that regorafenib for glioblastoma patients at first relapse led to a significant longer overall survival, compared to the control group treated with lomustine (7.4 vs. 5.6 months, P = 0.0009; hazard ratio 0.5) [4]. On the other hand, grade 3 or 4 side effects occurred more frequently in patients treated with regorafenib than in patients receiving lomustine [4]. Reported grade 3 and 4 laboratory abnormalities in patients treated with regorafenib included anemia, lymphocytopenia, neutropenia, decreased platelet count, hypertransaminasemia, increased blood bilirubin, and increased serum amylase and lipase [4–6]. The most frequent grade 3 and 4 clinical adverse events were hypertension, hand-foot skin reaction, fatigue, and diarrhea [4–7]. Nevertheless, regorafenib did not negatively affect health-related quality of life in the REGOMA trial compared to the control group treated with lomustine [8].

However, to date limited data exist on the use of regorafenib in pretreated WHO CNS grade 3 and 4 glioma patients at progression in a real-life setting, i.e., at the first relapse or after at least two or more treatment lines including standard therapy options before regorafenib initiation. Therefore, we conducted a retrospective single-center study to evaluate the efficacy concerning survival and side effects of regorafenib in this group of patients.

## Patients and methods

### Patients

Between December 2018 and November 2021, patients were retrospectively selected for evaluation if (i) they had a histomolecularly defined WHO CNS grade 3 or 4 glioma according to the recent fifth edition of the WHO Classification of Tumors of the Central Nervous System (2021) [9], (ii) radiologically confirmed tumor relapse according to the criteria defined by the Response Assessment in Neuro-Oncology (RANO) Working Group [10] before initiation of regorafenib, and (iii) at least one cycle of regorafenib was completed.

We collected clinical characteristics, i.e., survival, tumor characteristics such as the methylation status of the O^6^-methylguanine-DNA methyltransferase (MGMT) promoter, type and number of pretreatment lines, number of regorafenib cycles, MRI changes before and during regorafenib, laboratory abnormalities, and adverse clinical effects.

### Regorafenib therapy

At diagnosis of tumor relapse according to the RANO criteria, regorafenib was administered according to the REGOMA trial with regorafenib 160 mg once daily for the first 3 weeks of each four-week cycle with individual dose adjustment depending on side effects [4]. Regorafenib therapy was not administered within a clinical trial, but in the context of a salvage therapy (i.e., based on the patient’s individual decision when all other standard therapies were exhausted), and was also recommended by the local interdisciplinary neurooncological tumor board, especially when all conventional treatment options were no longer available. Treatment monitoring and follow-up was performed as part of routine clinical care and included a weekly differential blood count, laboratory testing of liver and renal function every 2–4 weeks, and an electrocardiogram monthly. Regorafenib-related side effects were evaluated according to the Common Terminology Criteria for Adverse Events (CTCAE; version 5.0).

### Clinical and neuroradiological follow-up

After initiating off-label therapy with regorafenib, clinical evaluation was performed every 4–8 weeks, and contrast-enhanced MRI was obtained after every second cycle or in case of neurological deterioration. Tumor progression on MRI during regorafenib therapy was assessed according to the RANO criteria [10]. The treatment was discontinued in case of clinical or radiological disease progression, occurrence of unacceptable toxicity, or following the patient’s explicit wish.

### Outcome and regorafenib efficacy

Regorafenib efficacy was assessed using progression-free survival (PFS) and overall survival (OS) as outcome parameters. PFS and OS were calculated from the start of regorafenib until the date of tumor progression or death, respectively.

### Statistical analysis

Descriptive statistics are provided as mean and standard deviation or median and range. The student’s *t*-test was used to compare two groups when variables were normally distributed, and the Mann–Whitney *U* test was used if variables were not distributed normally. Survival analyses were performed using the log-rank test. P-values of 0.05 or less were considered significant. Statistical analyses were performed using GraphPad Prism (Release 9.1.2, GraphPad Software Inc.).

## Results

### Patient characteristics

Based on the search criteria, 30 patients with WHO CNS grade 3 or 4 gliomas treated with regorafenib at relapse were retrospectively identified. Twenty-six patients (87%) had a WHO CNS grade 4 glioma, and 4 patients (13%) had a WHO CNS grade 3 glioma. At relapse, the median Karnofsky performance status (KPS) was 80% (range 60–100%), and the median Eastern Cooperative Oncology Group (ECOG) performance score was 1 (range 0–2). The median number of relapses and treatment lines before initiation of regorafenib was 2 (range 1–4). The majority of patients (73%) had two or more pretreatment lines. The rate of patients with three and four prior lines of treatment was 27% and 13%, respectively. At first relapse, regorafenib was administered in 27% of patients. Patients’ characteristics and detailed pretreatment information are listed in Table 1.

**Table 1:** Characteristics of patients treated with regorafenib

Characteristic	n	%
Age (years)		
Median age	54 (range 30–70)	
< 40	2	7%
40–59	17	57%
≥ 60	11	37%
Sex		
Female	11	37%
Male	19	63%
Karnofsky performance status		
60%	1	3%
70–80%	17	57%
90–100%	12	40%
ECOG performance status		
0	12	40%
1	17	57%
2	1	3%
Neuropathology at initial diagnosis		
Glioblastoma, IDH-wildtype, WHO CNS grade 4^a^	24	79%
Astrocytoma, IDH-mutant, WHO CNS grade 4	2	7%
Astrocytoma, IDH-mutant, WHO CNS grade 3	2	7%
Oligodendroglioma, IDH-mutant, 1p/19q-codeleted, WHO CNS grade 3	2	7%
MGMT promoter		
Methylated	16	53%
not methylated	14	47%
First-line treatment	30	100%
Resection or biopsy, RT with concomitant and adjuvant TMZ	22	73%
CR	13	43%
PR or biopsy	9	30%
Tumor-treating fields	4	13%
Resection or biopsy, chemoradiation with TMZ plus CCNU	5	17%
CR	3	10%
PR or biopsy	2	7%
Resection (CR), RT alone	2	7%
Experimental therapy^a^	1	3%
Second-line treatment	22	73%
CCNU-based chemotherapy	8	27%
Resection, RT, adjuvant CCNU-based chemotherapy	4	13%
Resection, RT with concomitant and adjuvant TMZ	3	10%
Resection, adjuvant TMZ	3	10%
Resection, adjuvant CCNU-based chemotherapy	2	7%
TMZ monotherapy	1	3%
RT alone	1	3%
Third-line treatment	8	27%
CCNU-based chemotherapy	2	7%
Resection, RT, adjuvant CCNU-based chemotherapy	1	3%
Resection, RT alone	1	3%
Resection, adjuvant CCNU-based chemotherapy	1	3%
RT, adjuvant CCNU-based chemotherapy	1	3%
Proton-RT, adjuvant TMZ	1	3%
TMZ monotherapy	1	3%
Fourth-line treatment	4	13%
Bevacizumab	2	7%
CCNU-based chemotherapy	2	7%

*AGK* acylglycerol kinase; *BRAF* v-Raf murine sarcoma viral oncogene homolog B; *CCNU* lomustine; *CR* complete resection; *ECOG* eastern cooperative oncology group; *IDH* isocitrate dehydrogenase; *MGMT* O^6^-methylguanine-DNA methyltransferase; *PR* partial resection; *RT* fractionated radiotherapy; *TMZ* temozolomide

^a^one glioblastoma patient had a AGK-BRAF gene fusion;

^b^Resection (CR) followed by RT with concomitant TMZ, adjuvant therapy with palbociclib and tumor-treating fields

### Regorafenib-related side effects

Overall, a total number of 94 regorafenib cycles were applied entirely. The median number of cycles applied per patient was 2 (range 1–9 cycles). Due to side effects, in 9 patients (29%), the regorafenib dose was reduced to 120 mg. Regorafenib-related side effects were not significantly increased in patients with two or more treatment lines than patients who received regorafenib at first relapse (55% vs. 50% grade 3 and 4 side effects; P > 0.05).

**Fig. 1:**
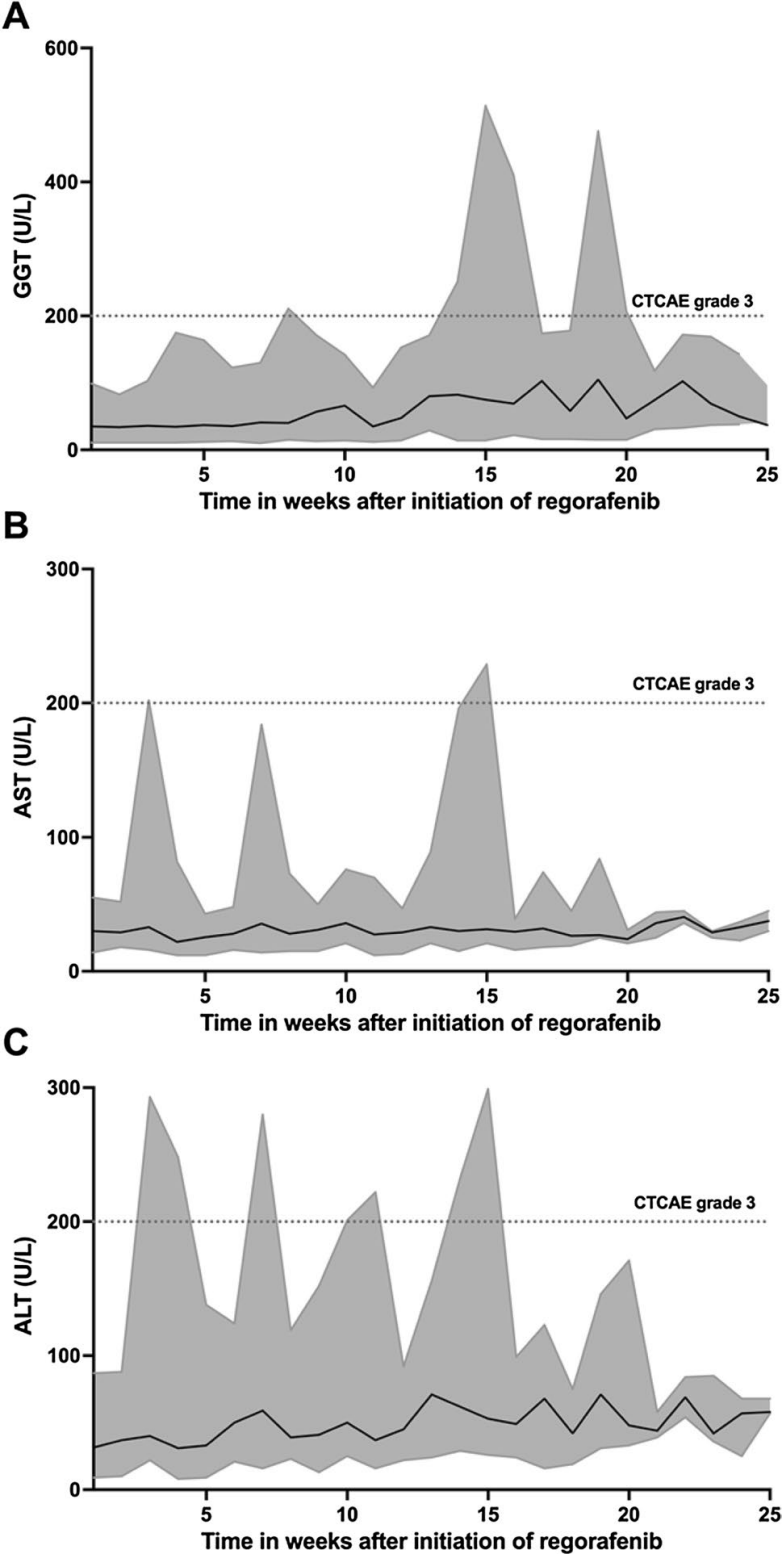
Averaged laboratory findings of all patients over the course of 25 weeks after initiation of regorafenib displayed as median (black line) and range (grey area). The onset of grade 3 toxicity with increased gamma-glutamyltransferase (GGT) and aspartate aminotransferase (AST) was more common at a later phase of regorafenib therapy (**A, B**) than grade 3 toxicity with increased alanine aminotransferase (ALT) (**C**)

A total number of 321 blood tests were evaluated. In 16 patients (53%), grade 3 or 4 laboratory abnormalities were observed. Grade 4 toxicity occurred in two patients (7%) and consisted of lymphocytopenia, hypertransaminasemia, and increased gamma-glutamyl transferase (GGT). The most frequent grade 3 laboratory abnormalities were lymphocytopenia and hypertransaminasemia in 6 patients (20%) and increased lipase in 5 patients (17%). The evolution of the aspartate aminotransferase (AST), alanine aminotransferase (ALT), and GGT laboratory abnormalities during regorafenib is shown in Fig. 1. Further details of laboratory abnormalities are listed in Table 2.

**Table 2:** Regorafenib-related toxicity

CTCAE term	Grade 1 *n* (%)	Grade 2 *n* (%)	Grade 3 *n* (%)	Grade 4 *n* (%)	Any grade *n* (%)
Laboratory abnormalities					
White blood cell decreased	7 (23%)	5 (17%)	1 (3%)	0 (0%)	13 (42%)
Neutrophil count decreased	5 (17%)	3 (10%)	1 (3%)	0 (0%)	9 (30%)
Lymphocyte count decreased	6 (20%)	10 (33%)	6 (20%)	1 (3%)	23 (77%)
Platelet count decreased	2 (7%)	3 (10%)	0 (0%)	0 (0%)	5 (17%)
Hemoglobin decreased	12 (40%)	1 (3%)	1 (3%)	0 (0%)	14 (47%)
Lipase increased	5 (17%)	1 (3%)	5 (17%)	0 (0%)	11 (37%)
ALT/AST increased	9 (30%)	0 (0%)	6 (20%)	1 (3%)	16 (53%)
GGT increased	5 (17%)	6 (20%)	2 (7%)	1 (3%)	14 (47%)
Clinical adverse effects					
Weight loss	0 (0%)	0 (0%)	2 (7%)	0 (0%)	2 (7%)
Hand-foot skin reaction	0 (0%)	0 (0%)	4 (13%)	0 (0%)	4 (13%)
Skin rash	0 (0%)	0 (0%)	1 (3%)	0 (0%)	1 (3%)

*ALT* alanine aminotransferase; *AST* aspartate aminotransferase; *CTCE* common terminology criteria for Adverse Events by the National Cancer Institute (version 5.0); *GGT* gamma-glutamyltransferase

No grade 4 clinical adverse effects were observed in the present study. The most frequent grade 3 clinical adverse effects were hand-foot skin reactions (n = 4 patients) and weight loss (n = 2 patients). Hand-foot skin reactions occurred after the first two cycles in three patients and after 8 cycles in one patient. All clinical adverse effects are listed in Table 2.

### Regorafenib efficacy in terms of outcome

**Fig. 2:**
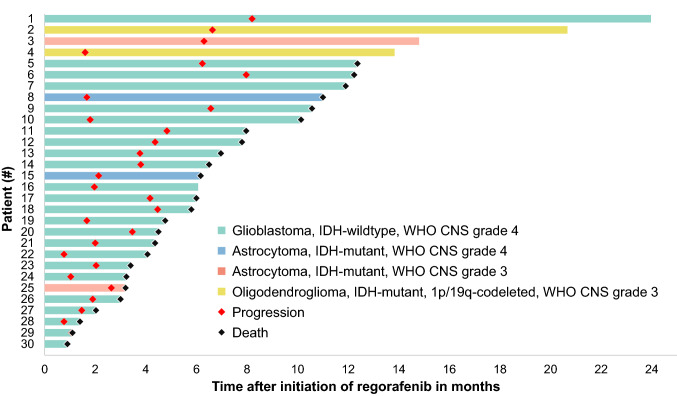
Swimmer plot of 30 patients with glioma at relapse treated with regorafenib sorted by overall survival after initiation of therapy. Time to progression ranged from 0.8 to 8.2 months. Patients with oligodendroglioma (#2, 4) were alive after 13.8 and 20.7 months, respectively. Most patients with glioblastoma (96%) and astrocytoma (75%) had died

**Fig. 3:**
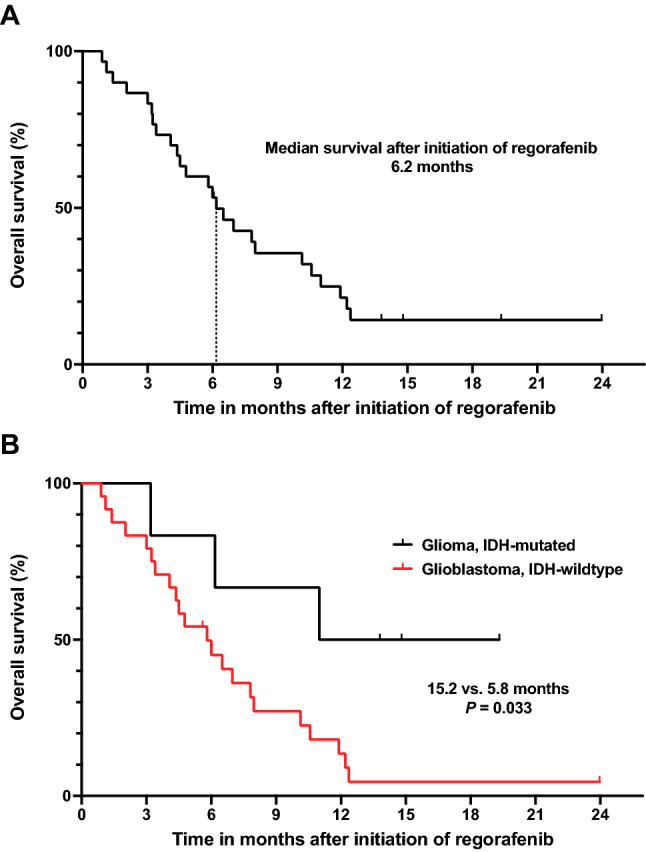
Kaplan–Meier plots for the median overall survival of all patients (**A**), and patients stratified according to the IDH-mutation status (**B**). Patients with an IDH-mutant glioma had a significantly 2.6-fold longer overall survival than patients with an IDH-wildtype (i.e., glioblastoma) (15.2 vs. 5.8 months; P = 0.033)

At the time of data evaluation, all patients had discontinued regorafenib therapy. Twenty-five patients had died (83%), and 5 patients (17%) were still alive. No patient was lost on follow-up. The median PFS was 2.6 months (range 0.8–8.2 months), and the PFS rate at 6 months was 23%. The median OS was 6.2 months (range 0.9–24 months), and the rate of OS at 6 months was 57%. One year after initiation of regorafenib treatment, progression had occurred in all patients, and the OS rate was 20%. Figure 2 provides the patient outcome from the regorafenib start to progression or death. Patients with IDH-mutated glioma had a significantly 2.6-fold longer median OS than patients with IDH-wildtype glioma (15.2 vs. 5.8 months; P = 0.033) (Fig. 3).

In patients with multiple pretreatments compared to patients who received only first-line therapy prior to recurrence, there was no significant difference in PFS (3.2 vs. 1.9 months; P > 0.05) and OS (6.9 vs. 4.4 months; P > 0.05). In the subgroup of patients with IDH-wildtype glioblastoma, OS was also not significantly different in patients with two or more pretreatments than patients who had received first-line treatment only (6.5 vs. 4.4 months; P > 0.05). Patients with a MGMT promoter methylation (n = 16; 53%) had not a significantly longer OS than patients without promoter methylation (6.5 vs. 5.3 months; P > 0.05). Likewise, a Karnofsky performance status of 90% or higher in the group of glioblastomas was not associated with a longer OS (7.8 vs. 4.5 months; P > 0.05). The median OS of patients that experienced grade 3 or 4 side effects was 7.9 months compared to 4.1 months for patients with grade 1 or 2 side effects (P > 0.05).

Of the two patients with oligodendroglioma and favorable outcome (OS > 12 months) despite early regorafenib discontinuation, one patient received 6 cycles of temozolomide chemotherapy (150–200 mg/m^2^ on day 1–5 of a 28-day cycle) after regorafenib (patient #2, Fig. 2). The other patient received no further treatment after regorafenib was discontinued and follow-up examinations over one year showed no progression (patient #4, Fig. 2).

## Discussion

The main finding of the present study is that not only glioblastoma patients at first relapse—as suggested by the REGOMA trial—but also patients with progressive WHO grade 3 or 4 gliomas, predominantly with two pretreatment lines or more, benefit from regorafenib concerning OS despite considerable grade 3 or 4 side effects in more than the half of the patients.

In our study, the OS of patients treated with regorafenib was broadly comparable with the REGOMA trial [4]. Although most patients had two or more pretreatment lines, the OS of 6.2 months was only slightly shorter than the OS in the REGOMA trial (7.4 months) [4]. A recent retrospective single-center study evaluating 54 glioblastoma patients reported a considerably longer OS of 10.2 months [11]. The longer OS in that study compared with our results may be explained best by selecting patients who had received first-line therapy only and had a better ECOG performance score of ≤ 1 [11].

In contrast, Tzaridis et al. reported an OS of 4.2 months in 24 patients with recurrent glioma (fraction of IDH-wildtype gliomas, 79%) [12]. In addition, Zeiner and colleagues observed a slightly shorter median OS of 3.2 months in 21 glioma patients at relapse (fraction of IDH-wildtype gliomas, 71%) [13]. Compared with our results, the shorter OS in the latter two studies may be partially related to a worse clinical condition before regorafenib initiation. In the study by Tzaridis et al. the KPS in more than half of the patients (63%) was not more than 70%, and 25% of patients had a KPS of 50% or 60% [12]. Similarly, in the study of Zeiner et al. the median KPS was 70% (range 50–100%) [13]. In contrast, in approximately 80% of patients in the present study, the KPS was 80% or even higher. A small series by Kebir and colleagues reported a median PFS of 3.5 months in 6 patients with recurrent glioma. However, data on the OS were not reported [14].

When comparing the efficacy of regorafenib with lomustine, which is commonly used to treat glioma patients at relapse, it is essential to discuss the survival data compared to other clinical trials. In particular, the OS of 7.4 months reported for patients treated with regorafenib in the REGOMA trial was shorter than in other trials using lomustine in the control arm for the treatment of progressive glioblastoma patients such as the REGAL and the EORTC 26101 trial, with an OS of 9.8 and 8.6 months, respectively [4, 15, 16]. In contrast, the OS of the lomustine control group in the REGOMA trial was only 5.6 months, which may have contributed to the significant survival benefit for regorafenib.

However, the reasons for this discrepancy are still unclear. One could argue that differences in clinical and prognostic factors could be an explanation. Indeed, the rate of patients on glucocorticoids at baseline was higher in the REGOMA trial (62%) than in the REGAL and EORTC 26101 trial (40% and 48%, respectively) [4, 15, 16]. In addition, patients in the REGOMA trial were slightly older (median age, 59 vs. 54 years), and fewer patients had a KPS of ≥ 90% (47% vs. 63%) compared to the REGAL trial [4, 16]. One aspect that may further limit the value of such a cross-trial comparison is that in the REGAL trial, the information on both the IDH mutation status and MGMT promoter methylation was not available [16]. In addition, in the EORTC 26101 trial the information on MGMT promoter methylation status was available only in half of the patients [15]. Nevertheless, the REGOMA trial was a randomized clinical trial, and the equal distribution of patients with poorer prognostic factors in both treatment arms after randomization may explain the shorter OS compared to other trials [17].

On the other hand, it remains unclear why in the retrospective study by Lombardi et al. the OS is considerably higher than in the REGOMA trial, although there were no relevant differences in terms of age (55 vs. 55 years), ECOG performance status (≤ 1 in both studies), the fraction of IDH-wildtype gliomas (95% vs. 91%), and pretreatment (all patients received temozolomide chemoradiation in both studies) [4, 11]. A possible explanation might be the retrospective character and a selection bias related to non-randomization. Notwithstanding, besides known prognostic factors, parameters that remain to be determined may be relevant for the response to regorafenib.

Furthermore, in contrast to the studies evaluating regorafenib in lomustine-naive patients at first relapse [4, 11], the vast majority of patients in the present study (90%) and in earlier published studies [12–14] had already received lomustine. Thus, our findings suggest that regorafenib is an effective treatment option in most patients for whom alkylating chemotherapy is no longer an option.

In line with earlier studies [4, 11], a methylated MGMT promoter did not affect the efficacy of regorafenib in the present study and may, therefore, also be a promising treatment option in patients with an unmethylated MGMT promoter. Accordingly, regorafenib is currently under investigation in patients with newly diagnosed glioblastoma with unmethylated MGMT promoter in the GBM AGILE trial [18].

In terms of tolerability, our results show that a high percentage of patients experienced drug-related adverse events. Nevertheless, although 73% of the patients had two or more pretreatment lines in the present study, the rate of grade 3 or 4 events did not exceed the rate in patients treated in the REGOMA trial (53% and 56%, respectively) [4]. On the other hand, more patients required dose reductions, with 27% of the patients in the present study compared to the REGOMA trial (17%) [4]. However, Lombardi and colleagues reported an even higher rate of 37% in their retrospective monocentric study [11]. Overall, our results suggest that patients with multiple previous treatment lines do not more frequently experience side effects with consecutive dose reductions.

In summary, regorafenib is a promising treatment option currently under clinical investigation in a prospective multicenter trial. Our results suggest that regorafenib is an effective therapy for patients with recurrent WHO CNS grade 3 or 4 gliomas at a later stage of the disease despite considerable grade 3 or 4 side effects. To validate our results, controlled clinical trials are needed to evaluate regorafenib especially in patients with recurrent WHO CNS grade 3 glioma or patients with multiple previous lines of treatments.
